# Using case management in a universal health coverage system to improve quality of life of frequent Emergency Department users: a randomized controlled trial

**DOI:** 10.1007/s11136-017-1739-6

**Published:** 2017-11-29

**Authors:** Katia Iglesias, Stéphanie Baggio, Karine Moschetti, Jean-Blaise Wasserfallen, Olivier Hugli, Jean-Bernard Daeppen, Bernard Burnand, Patrick Bodenmann

**Affiliations:** 1 School of Health Sciences (HEdS-FR), University of Applied Sciences Western Switzerland (HES-SO), Route des Cliniques 15, 1700 Fribourg, Switzerland; 2 Center for the Understanding of Social Processes, University of Neuchâtel, Neuchâtel, Switzerland; 3 Life Course and Social Inequality Research Center, University of Lausanne, Lausanne, Switzerland; 4 Institute of Social and Preventive Medicine, Lausanne University Hospital, Lausanne, Switzerland; 5 Health Technology Assessment Unit, Lausanne University Hospital, Lausanne, Switzerland; 6 IEMS Plateforme interfacultaire en économie et management de la santé, University of Lausanne, Lausanne, Switzerland; 7 Health Technology Assessment Unit, Lausanne University Hospital, 1011 Lausanne, Switzerland; 8 Emergency Department, Lausanne University Hospital, Lausanne, Switzerland; 9 Alcohol Treatment Center, Lausanne University Hospital, Lausanne, Switzerland; 10 Vulnerable Population Unit, Department of Ambulatory Care and Community Medicine, Lausanne University Hospital, Lausanne, Switzerland

**Keywords:** Vulnerable population, WHOQOL, Environment, Mental health, Physical health, Social relationship

## Abstract

**Purpose:**

Frequent Emergency Department users are likely to experience poor quality of life (QOL). Case management interventions are efficient in responding to the complex needs of this population, but their effects on QOL have not been tested yet. Therefore, the aim of our study was to examine to what extent a case management intervention improved frequent Emergency Department users’ QOL in a universal health coverage system.

**Methods:**

Data were part of a randomized controlled trial designed to improve frequent Emergency Department users’ QOL at the Lausanne University Hospital, Switzerland. A total of 250 frequent Emergency Department users (≥ 5 attendances during the previous 12 months) were randomly assigned to the control (*n* = 125) or the intervention group (*n* = 125). The latter benefited from case management intervention. QOL was evaluated using the WHOQOL-BREF at baseline, two, five and a half, nine, and twelve months later. It included four dimensions: physical health, psychological health, social relationship, and environment. Linear mixed-effects models were used to analyze the change in the patients’ QOL over time.

**Results:**

Patients’ QOL improved significantly (*p* < 0.001) in both groups for all dimensions after two months. However, environment QOL dimension improved significantly more in the intervention group after 12 months.

**Conclusions:**

Environment QOL dimension was the most responsive dimension for short-term interventions. This may have been due to case management’s assistance in obtaining income entitlements, health insurance coverage, stable housing, or finding general health care practitioners. Case management in general should be developed to enhance frequent users’ QOL.

Trial registration: http://www.clinicaltrials.gov, Unique identifier: NCT01934322

## Introduction

Frequent Emergency Department (ED) users are a small heterogeneous group of patients who often visit the ED (i.e., five or more visits per year [1]). They represent between 3 and 8% of ED’s patients, but account for 12–28% of all ED visits, often overcrowding the ED [2–4]. The high number of visits of such a small number of patients leads to concerns about the appropriate use of ED resources [5, 6] and its consequences on health care costs [7, 8]. Therefore, this subgroup of patients is of great interest for interventions aiming at improving its management within and outside the ED [9, 10]. Initially, the ED role in the health care system was to treat life-threatening conditions but has ultimately changed since the delivery of acute care has gradually shifted from general practitioners to EDs [11]. Acute care may not always meet frequent ED users’ specific needs, as these needs are more social- or psychosocial-oriented (e.g., social health insurance, stable housing). Furthermore, these patients frequently do not know how to navigate the health care system.

Frequent ED users are considered vulnerable patients, meaning, they are more at risk of having poor social, physical, and psychological health [12], not only because of their singular health care consumption of ED, but also their use of hospitalization, outpatient visits, primary care practitioners, social workers, and psychiatrists [13–16]. They are also vulnerable because they are likely to be isolated [8], to have a low socio-economic status [9, 17, 18], to suffer from substance abuse [4, 17, 19], and, in general, to have a worse health status (e.g., chronic diseases [4, 8, 20], mental health issues [8, 10, 18]) and a higher rate of morbidity and mortality than average [14, 16, 18]. Case management, the most frequently tested intervention [21], has been described as responding to some of the complex needs of frequent ED users: it reduces drug use [22], homelessness [23–25], and improves social and clinical outcomes [21, 26, 27]. Case management does not only focus on acute care, but also on coordination and organization of care, on guiding patients through the healthcare system, and on providing social support inside the hospital and also often [26].

Vulnerable people are also more likely to experience poorer quality of life (QOL) than non-vulnerable people [28, 29]. QOL is defined by the World Health Organization (WHO) as an individual’s perception of several life domains such as physical health, psychological state, level of independence, social relationships, personal beliefs, and relationships. This multidimensional concept is conceived as a measure of societal progress and is not merely defined by an absence of infirmity or disease, but by physical, mental, and social well-being [30, 31]. It includes both positive and negative aspects of life domains in interaction with the patient’s environment [32]. The frequent ED users’ QOL is considered as a major issue to take into account to fulfill the specific needs of this population [9, 22], but studies which address this topic remain rare. A review of the existing literature revealed that only one study focused on frequent ED users’ QOL [33], and the intervention program in the study did not significantly influence patients’ QOL. There were several shortcomings (convenient sample, short follow-up) in that study and therefore further studies are warranted to determine the true benefits of an intervention [34].

Our study’s goal is to fill this gap in the literature using a randomized controlled trial (RCT). First, we evaluated the initial level of frequent ED users’ QOL and compared it to a population norm. Second, we analyzed the impact of a case management intervention on frequent ED users’ QOL within a universal health coverage system and compared the intervention to standard emergency care.

## Method

### Design

We conducted a RCT with two parallel groups to compare the impact of a case management intervention to standard emergency care on three outcomes: the first outcome, the number of ED visits [35], and the second outcomes, QOL, and cost (more detailed information can be found in the published protocol [36], as sample size calculation information). The RCT took place at Lausanne University Hospital ED, which is a reference hospital in the French-speaking part of Switzerland providing medical, surgical, and mental health care and counting 35,000 annual ED visits. It is one of the five teaching university hospitals located in Switzerland. In Switzerland, the number of ED visits was estimated to 20 visits per 100 inhabitants [37]. This number is close to other European countries (France and UK), whereas it is higher in USA (41 visits per 100 inhabitants) [37]. There is a higher density of EDs per square mile in Switzerland compared to France and USA enabling the Swiss population to rapidly access to an ED within close proximity. Another Swiss specificity is its low-volume ED (half of Swiss EDs saw less than one patient per hour). Consequently, critical equipment and imaging capabilities are less common in low-volume EDs and, when present, took longer to obtain, as well as consultations with specialists. This last feature does not characterize the Lausanne University Hospital ED.

### Patient selection and randomization

Eligible participants were frequent ED users, defined as visiting ED five or more times per year [1]. They also had to be at least 18 years old; to be able to communicate in French, German, Italian, English, or Spanish or through a community interpreter; to be able to provide informed consent; not to be incarcerated; not to have been in previous contact with any members of the case management team of Lausanne University Hospital; not to have a family member enrolled in the study; or to have a projected life expectancy lower than 18 months or to plan to leave Switzerland for the next 18 months.

The recruitment was conducted between May 2012 and July 2013, and participants were monitored during 12 months. Frequent ED users were identified using an automatic ED patient tracking software. During this period, which covered 24 h a day/7 days a week visits of frequent ED users, 1145 frequent ED users were identified. 928 (81%) were contacted by a nurse of the case management team, either during their visit to the hospital or by phone within 72 h after discharge. All the patients coming to the hospital had to give a phone number where they could be contacted during their hospital admission (e.g., their phone number, a family member’s phone number, or a friend’s phone number). Of the 928 patients contacted, 26.9% consented to participate in the study, 29.7% declined, 18.4% were unreachable (e.g., wrong phone number, did not answer the phone), and 24.9% had at least one exclusion criterion. Those who refused to participate did not differ in sex or nationality, but were older than enrolled participants (*p* = 0.030). A total of 250 participants were included in the study by a nurse of the case management team.

During the first visit, a nurse of the case management team explained the study to the participants. Once the participants signed the consent form, they completed an hour and a half assessment, which included baseline demographic characteristics, social determinants of health, mental and somatic diseases, risk behaviors, and QOL with a nurse of the case management team. Once the data were collected, a statistician randomly assigned the participants to either the intervention group or to the control group. After the assignment, the case management team and the research team could not be blinded to the patient’s allocation due to their activities and contacts. The study included a follow-up at two, five and a half, nine, and twelve months after the first assessment by a study nurse of the research team. She evaluated, among other variables, the QOL of the participants. Among the 250 participants included at baseline, 193 (99 persons in the control group and 94 in the intervention group) were still in the study at the last follow-up. Of the 57 patients who dropped out (22.8% of the sample), 20 died during the study (10 in each group) and 37 (16 in the control group and 21 in the intervention group) did not participate to the whole study. On the 37 patients who did not participate to the whole study, five left Switzerland (two in the control group and three in the intervention group), 10 refused to continue in the study (three and seven, respectively), and the remaining 22 were lost to follow-up (12 and 10, respectively). For further details on when patients dropped out during the study for each group, see Table 1.

**Table 1:** Sample size by assessment time of QOL

	Available sample size			Deceased (*n* = 20)			Lost to follow-up (*n* = 37)		
	Sample	Ctrl	Itv	Sample	Ctrl	Itv	Sample	Ctrl	Itv
Time assessment of QOL									
At baseline	250	125	125	0	0	0	0	0	0
At 2 months	219	102	101	4	2	2	27	21	22
At 5.5 months	200	97	94	11	5	6	39	23	25
At 9 months	198	102	94	18	10	8	34	13	23
At 12 months	193	99	94	20	10	10	37	16	21

*Ctr* control group, *Itv* intervention group, *QOL* quality of life

### Case management intervention

The frequent ED users in the control group received standard emergency care (through ED, specialists, physicians, and nurses focused on somatic and/or mental diseases and/or behavioral specific acute problems). They also received information about the case management program and were eligible for services following the completion of the study if desired. In the intervention group, in addition to standard emergency care, participants received a case management intervention (coordination of care, not only focused on acute care) at baseline, one, three, and five months by a nurse (a member of the case management team). Furthermore, the participants had the opportunity to contact, at any moment, one of the members of the case management team in an “open-door policy perspective” (for full details see [36]). The case management team was made up of four nurse practitioners and a chief resident (who coordinated care and facilitated communication inside the health care system, indirectly involved with patients of the intervention groups). All the members of the case management team were trained to case management and received intensive training in motivational interviewing. First, the team brought counseling to the patients based on motivational interviewing and cross-cultural competencies to identify the social determinants of patients’ health and their use of medical services. Second, it provided concrete assistance in obtaining income entitlements, better health insurance coverage, stable housing, and educational opportunities for the participants. Finally, the team referred patients to a mental health department, substance abuse services, or a new general care provider if necessary. The contact between participants and the case management team during the intervention was face to face or at least by phone.

The case management team provided individualized services to each participant of the intervention group. Several “vulnerability experts” from different departments in the hospital such as psychiatrists, alcohol specialists, and social workers supported the team to provide the more adapted services. The central point of the intervention was to establish a network between providers and services at the hospital level, as well as at the community level, to promote continuity within the care and thus improve the use of the health care system.

During the study, no participants of the control group asked for case management intervention and all the participants of the intervention group who completed the WHOQOL at baseline and follow-up received the full intervention.

### Measures

#### QOL

 QOL was evaluated with the French version of WHOQOL-BREF [38] at baseline, two, five and a half, nine, and 12 months later (validated version of the WHOQOL-BREF was also available for participants speaking German, Italian, English, and Spanish). The questionnaire was based on satisfaction questions across four domains of quality of life: physical health (7 items: pain and discomfort; energy and fatigue; sleep and rest; dependence on medication; mobility; activities of daily living; working capacity), psychological health (6 items: positive feelings; negative feelings; self-esteem; thinking, learning, memory, and concentration; body image; spirituality, religion, and personal beliefs), social relationships (3 items: personal relations; sex; practical social support), and environment (8 items: financial resources; information and skills; recreation and leisure; home environment; access to health and social care; physical safety and security; physical environment; transport). Each question was rated in reference to the last 2 weeks using a five-point scale. A percentage rating within each domain was computed with scores ranging from 0 (lowest QOL) to 100 (highest QOL) as define by the instrument use.

#### Health status

A health care practitioner assessed health determinants using the WHO framework [17, 39]. For each participant, it was assessed if he or she had any (1) social issues (e.g., no insurance, no housing, social isolation, difficult familial situation, and difficult financial situation); (2) somatic issues (e.g., chronic disease, complex medical treatment, somatic polymorbidity, and physical disability); (3) mental health issues (e.g., psychiatric polymorbidity, post-traumatic stress, and psychological development disorders); and (4) behavioral issues (e.g., substance use, sexually risky behaviors, and interpersonal violence). For each group of issues, its absence was coded 0 and the presence of at least one issue was coded 1 (for the whole list of issues assessed, see [40]).

#### Covariates

Socio-demographic information was also collected: age, gender, nationality, level of education, and level of spoken French. Socio-demographic and health information were gathered and assessed during face-to-face interviews. According to the participants’ choice, QOL was assessed in face-to-face interviews or using a written questionnaire.

### Statistical analysis

#### Data analysis

First, descriptive statistics (frequencies and percentages for categorical data, means and standard deviations for quantitative data) were computed for each group and for the whole sample at baseline. We computed bivariate associations to detect differences between groups at baseline (Pearson χ^2^-test, Fisher’s exact tests, *t* tests, and Mann–Whitney tests depending on the distributions of the variables). Second, an analysis of RCT dropouts, composed of participants who died or those who did not participate in the whole study, was also performed with *t* test comparing levels of QOL between types of dropout participants and participants who completed the whole study at baseline. Third, we compared the level of QOL of the frequent ED users to specific values coming from a general adult French population [41] using one-sample *t* tests at baseline and after 12 months. The following values were chosen as norm for the analyses [41]: physical health: 76.9; psychological health: 67.0; social relationship: 74.5. No information of mean level of environment’s QOL was available. Fourth, we plotted the computed differences of QOL by dimension (QOL at 12 months minus QOL at baseline) and means and SD by groups to briefly describe the change in QOL over time (only possible for those participating at baseline and at 12 months, *n* = 193). Fifth, to test the impact of the intervention on original QOL measures (baseline compared to at two months, at five and a half months, at nine months, and at 12 months), linear mixed-effects models with participants as a random effect were run to analyze the changes over time in each dimension of QOL. The models were tested on the available information (250 participants at baseline, 219 at 2 months, 200 at five and a half months, 198 at nine months, and 193 at 12 months). The effects of time, group, and their interaction were tested, controlling for socio-demographic characteristics (age, gender, nationality, level of education, and spoken language) and health-related variables (number of social, somatic, mental health, and behavioral problems).

All analyses were carried out using STATA Data Analysis and Statistical software (version 12, StataCorp).

## Results

The sample’s characteristics are shown in Table 2. Participants were on average 46.1 ± 18.9 years old; 57% were men; 48% Swiss. Levels of QOL dimensions ranged on average between 53.0 and 58.0. There were no significant differences between intervention and control group at baseline, except for the level of education, with more non-response in the control group.

**Table 2:** Respondents’ characteristics and quality of life

	All (*n* = 250)	Intervention group (*n* = 125)	Control group (*n* = 125)	*p* Value of test^a,b^
Age^a^	46.1 (18.9)	46.0 (18.6)	46.3 (19.2)	0.891
Gender^b^				
Female	107 (42.8)	55 (44.0)	52 (41.6)	
Male	143 (57.2)	70 (56.0)	73 (58.4)	0.701
Country of origin^b^				
Switzerland	119 (47.8)	58 (46.4)	61 (49.2)	
Other European countries	44 (17.7)	24 (19.2)	20 (16.1)	
Non-European country	86 (34.5)	43 (34.4)	43 (34.7)	0.804
Level of education^b^				
Obligatory schooling	64 (25.6)	53 (42.4)	11 (8.8)	
High school, vocational school	113 (45.2)	49 (39.2)	64 (51.2)	
University, under graduate college	42 (16.8)	17 (13.6)	25 (20.0)	
Non-applicable, non-response	31 (12.4)	6 (4.8)	25 (20.0)	< 0.001
Spoken language^b^				
French without difficulty	203 (81.2)	102 (81.6)	101 (80.8)	
French with difficulty/other language	47 (18.8)	23 (18.4)	24 (19.2)	0.871
Presence of objective health issues				
Social issues^b^	182 (72.8)	93 (74.4)	89 (71.2)	0.570
Somatic issues^b^	173 (69.2)	90 (72.0)	83 (66.4)	0.338
Mental health issues^b^	126 (50.4)	62 (49.6)	64 (51.2)	0.800
Behavioral issues^b^	80 (32.0)	43 (34.4)	37 (29.6)	0.416
Quality of life at baseline^a^				
Physical health	53.5 (14.6)	54.1 (14.4)	52.9 (14.8)	0.517
Psychological health	53.0 (17.1)	52.4 (17.7)	53.6 (16.4)	0.561
Social relationships	57.8 (24.7)	58.1 (23.3)	57.5 (26.1)	0.857
Environment	57.8 (20.9)	56.9 (20.0)	58.6 (21.9)	0.542

^a^Means and standard deviations are reported. *T* tests were computed

^b^ *n* and percentages are given. Chi-square tests were computed

Participants who died (*n* = 20) during the study had the same level of QOL as those who participated in the whole study (physical health: *p* = 0.868; psychological health: *p* = 0.390; social relationship: *p* = 0.179; and environment: *p* = 0.380) at baseline. Dropout participants had the same level of QOL as participants who took part in the study in its entirety (physical health: *p* = 0.116; psychological health: *p* = 0.082; social relationship: *p* = 0.126) at baseline, except for environment with a lower mean level of 10 points (*p* = 0.006).

**Fig. 1:**
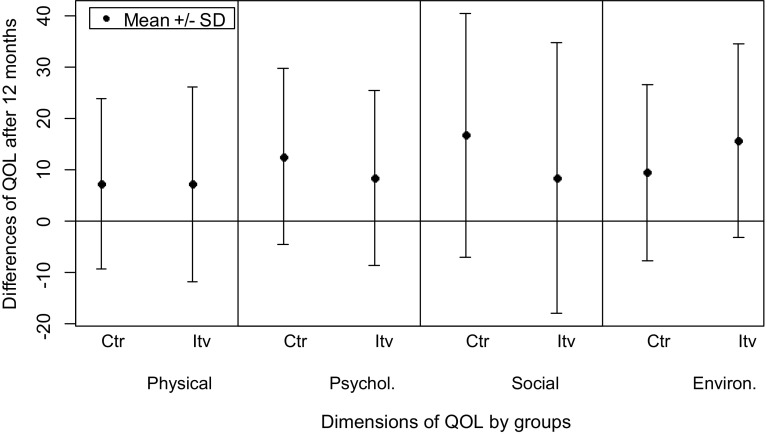
Differences of QOL after 12 months (QOL at 12 months minus QOL at baseline) by dimensions of QOL and by groups. *Ctr* control group, *Itv* intervention group, *Physical* physical health, *Mental* psychological health, *Social* social relationship, *Environ*. environment. Differences of quality of life (QoL) refer to QoL at 12 months minus QoL at baseline, with 0 meaning no changes over time and a value > 0 meaning an improvement over time

The mean levels of frequent ED users’ QOL at baseline were significantly lower than in the general population for three of the four dimensions (physical health: *t*(249) = − 25.4, *p* < 0.001; psychological health: t(249) = − 13.0, *p* < 0.001; social relationship: *t*(249) = − 10.7, *p* < 0.001; and environment: no available values for the general population). After 12 months, the mean level of QOL of frequent ED users, independently of the belonging group, compared to the general population was still significantly lower for physical health [*t*(192) = − 13.5, *p* < 0.001] and not any more different for the psychological health [*t*(192) = − 1.7, *p* = 0.087] and for the social relationship [t(192) = − 1.3, *p* = 0.213]. Before analyzing all the available data on QOL over time, we plotted the differences of QOL by dimensions for each group between the 12-month assessment and baseline for the 193 patients present during the whole study (QOL at 12 months minus QOL at baseline). Figure 1 shows that all the mean differences are greater than 0, which mean an improvement of QOL after 12 months.

The visual description of differences between baseline and 12-month follow-up was supported by the multivariate models (Table 3). It showed a significant improvement of QOL in both groups in the four dimensions considered at 12 months compared to baseline: an increase of 15% for physical health (*b* = 8.1, *p* < 0.001); an increase of 18% for psychological health (*b* = 9.7, *p* < 0.001); an increase of 24% for social relationship (*b* = 13.8, *p* < 0.001); an increase of 18% for environment (*b* = 10.4, *p* < 0.001). In addition, QOL already improved significantly in both groups after 2 months [only marginally for physical health (*p* = 0.069), with an increase of 5, 6, 6, and 7%, for the four aforementioned QOL dimensions, respectively]. Moreover, the environment quality of life dimension significantly improved more in the intervention group than in the control group with an increase of 18% for the control group and an increase of 28% for the intervention group after 12 months compared to the baseline (significant interaction: *b* = 5.8, *p* = 0.012).

**Table 3:** Analyses of impact of the intervention on original QOL measures (at baseline, two, five and a half, nine, and 12 months): linear mixed-effects models with participants as a random effect

	Quality of life dimensions (n = 250)											
	Physical health			Psychological health			Social relationships			Environment		
	Coef	95% CI	*p* Val	Coef	95% CI	*p* Val	Coef	95% CI	p Val	Coef	95% CI	*p* Val
M1												
Gr												
Intercept	52.9	[50.3 ; 55.5]	0.000	53.6	[50.6 ; 56.6]	0.000	57.5	[53.1 ; 61.9]	0.000	58.6	[54.9 ; 62.2]	0.000
Itv group	1.2	[− 2.5 ; 4.9]	0.528	− 1.3	[− 5.5 ; 3.0]	0.561	0.6	[− 5.7 ; 6.8]	0.858	− 1.6	[− 6.8 ; 3.6]	0.542
Time												
2 months	2.8	[− 0.2 ; 5.8]	0.069	3.3	[0.3 ; 6.3]	0.029	3.2	[− 1.2 ; 7.6]	0.159	4.1	[1.0 ; 7.2]	0.009
5.5 months	7.3	[4.2 ; 10.4]	0.000	6.3	[3.3 ; 9.4]	0.000	7.7	[3.1 ; 12.2]	0.001	9.5	[6.3 ; 12.6]	0.000
9 months	8.9	[5.9 ; 12.0]	0.000	9.9	[6.9 ; 13.0]	0.000	11.8	[7.3 ; 16.3]	0.000	11.2	[8.0 ; 14.4]	0.000
12 months	8.1	[5.0 ; 11.2]	0.000	9.7	[6.7 ; 12.8]	0.000	13.8	[9.2 ; 18.4]	0.000	10.4	[7.2 ; 13.6]	0.000
Time*gr												
2 months	0.1	[− 4.1 ; 4.4]	0.955	2.2	[− 2.0 ; 6.4]	0.304	1.8	[− 4.4 ; 8.1]	0.569	2.1	[− 2.2 ; 6.5]	0.343
5.5 months	− 3.8	[− 8.2 ; 0.6]	0.090	0.6	[− 3.7 ; 5.0]	0.785	0.5	[− 6.0 ; 6.9]	0.886	0.0	[− 4.5 ; 4.5]	0.990
9 months	− 1.3	[− 5.7 ; 3.1]	0.573	1.4	[− 3.0 ; 5.7]	0.542	2.0	[− 4.5 ; 8.5]	0.554	3.5	[− 1.0 ; 8.1]	0.128
12 months	− 0.7	[− 5.1 ; 3.7]	0.759	3.0	[− 1.4 ; 7.4]	0.175	0.1	[− 6.4 ; 6.6]	0.978	5.8	[1.3 ; 10.4]	0.012
M2												
Gr												
Intercept	65.1	[56.9 ; 73.4]	0.000	70.1	[60.1 ; 80.1]	0.000	81.2	[66.6 ; 95.8]	0.000	81.5	[70.2 ; 92.9]	0.000
Itv group	3.0	[− 0.8 ; 6.8]	0.121	0.5	[− 3.8 ; 4.7]	0.826	1.2	[− 5.1; 7.5]	0.709	0.1	[− 4.6 ; 4.8]	0.963
Time												
2 months	3.0	[− 0.8 ; 6.8]	0.079	3.3	[0.3 ; 6.2]	0.030	3.1	[− 1.3 ; 7.6]	0.168	3.7	[0.6 ; 6.7]	0.020
5.5 months	7.2	[4.1 ; 10.3]	0.000	6.3	[3.2 ; 9.3]	0.000	7.5	[3.0 ; 12.1]	0.001	9.2	[6.1 ; 12.4]	0.000
9 months	8.5	[5.4 ; 11.5]	0.000	9.2	[6.2 ; 12.3]	0.000	11.6	[7.0 ; 16.2]	0.000	11.0	[7.9 ; 14.2]	0.000
12 months	7.8	[4.7 ; 10.9]	0.000	9.4	[6.4 ; 12.5]	0.000	13.7	[9.2 ; 18.3]	0.000	10.2	[7.0 ; 13.4]	0.000
Time*gr												
2 months	0.2	[− 4.1 ; 4.4]	0.937	2.1	[− 2.0 ; 6.3]	0.315	1.9	[− 4.4 ; 8.2]	0.553	2.3	[− 2.0 ; 6.7]	0.298
5.5 months	− 3.9	[− 8.3 ; 0.5]	0.081	0.4	[− 3.9 ; 4.7]	0.865	0.4	[− 6.0 ; 6.9]	0.897	− 0.1	[− 4.6 ; 4.4]	0.969
9 months	− 1.0	[− 5.4 ; 3.4]	0.667	1.8	[− 2.6 ; 6.1]	0.425	2.0	[− 4.5 ; 8.5]	0.547	3.4	[− 1.2 ; 7.9]	0.146
12 months	− 0.5	[− 4.9 ; 3.9]	0.816	3.1	[− 1.3 ; 7.5]	0.163	0.0	[− 6.5 ; 6.5]	0.995	5.7	[1.2 ; 10.3]	0.013

*M1* model without control variables, *M2* model controlled for age, gender, nationality, level of education, and level of spoken French, number of social, somatic, mental health, and behavioral problems. Intercept—mean level of QOL for control group at baseline. *95% CI* 95% Confidence interval, *Coef*. regression coefficient, *p val* p value. Categories of reference—control group and baseline. *Gr*. group, *Itv* intervention group

## Discussion

There are four major findings in our study: lower level of QOL of ED frequent users compared to general population at baseline; a global increase in QOL already after two months; a higher increase of environment QOL dimension for frequent ED users in the case management intervention group after 12 months; and a non-significant effect of the case management intervention on physical health, psychological health, and social relationships QOL dimensions.

First, we evaluated the initial level of QOL of frequent ED users to assess their level of vulnerability. Compared to the general adult French population [41], the frequent ED users of this RCT showed a lower mean level of QOL in all dimensions: physical health, psychological health, and social relationships (no data were available for the environment dimension). These average levels of frequent ED users’ QOL were between 19 and 30% lower than the reference norms established by Baumann and his colleagues for a general French population (physical health: 23.7 points lower; psychological health: 12.9 points lower; and social relationships: 16.4 points lower). Thus, in line with previous studies [33], frequent ED users were more likely to experience poorer QOL than non-vulnerable people.

Second, after a couple of months in the study (two to five and half months), we observed a general improvement of the patients’ QOL, regardless of the dimensions of QOL considered and the intervention/control group. This general improvement may be explained by several factors. The first one is related to the study’s design itself, as all participants (whichever groups they belong to) were followed by a study nurse. Every 3 months, she contacted the frequent ED users by phone and asked them questions about their QOL. Thus, the study brought attention to this population, which can have had a positive impact on the patient’s QOL [42, 43]. Furthermore, as we already know from other studies, frequent ED users feel discriminated against [40, 44] and are considered as not having their place in the ED [45, 46]. Therefore, the presence and work of the study’s nurse could have provided them with a form of legitimacy, hence contributing to the improvement in their QOL. They may also have found social and emotional support in their interactions with the study’s nurse, a care professional with whom they could talk about their care (which takes a significant place in their life) and their well-being. The second reason which can explain the improvement of patients’ QOL is the phenomenon of regression to the mean. At baseline, the frequent ED users’ levels of QOL were fairly low, but also under the average level of QOL. At the end of the intervention, they may have naturally regressed to the mean, showing an improvement of patients’ QOL. However, this increase did not fully offset the initial difference found in comparison to the reference norms for a general French population for physical health with a mean still 19% lower than the norm (the remaining differences for psychological health (5%) and for social relationship (3%) were not significant anymore). Finally, being a frequent ED user may in fact have been a transitory state [47]. After 12 months, a certain number of frequent users are expected to get better and consequently to have a higher level of QOL. Overall, from a clinical point of view, healthcare practitioners should be aware that being available and listening creates a favorable environment for vulnerable patients. This may be an easy step to achieve a better health care and to enhance QOL without costly and time-consuming medical assistance.

The third central finding of our study is the higher positive impact of case management intervention on the frequent ED users’ environment QOL dimension. Part of the case management intervention was to focus on the environment QOL dimension by providing social assistance. Some of social-oriented services provided to frequent ED users were: obtaining income entitlements, health insurance coverage, stable housing, schooling for children, preventing potential violence in the home, and finding general health care practitioners or specialists. Indeed, the environment dimension measured by the WHOQOL is reflected in the indicators of financial resources, freedom, physical safety and security, accessibility and quality of health, and social cares, leisure, training, physical environment, and transport. Thus, the case management intervention improved the patient’s environment QOL dimension by specifically targeting the physical safety and security, the financial resources, and the access to health care.

The fourth finding of our study is a non-significant impact of the case management intervention on the three other dimensions of QOL assessed (physical health, psychological health, and social relationships). Two reasons can explain this result. First, the intervention did not directly target these dimensions compared to the environment one. With the psychological dimension, the intervention simply referred patients to mental health specialists (psychiatrist, psychologist), whereas psychological health QOL was measured through different aspects of mental health such as body image, self-esteem, negative feelings, and concentration. As far as the behavior dimension, the intervention directed the patients to substance abuse services and got them in touch with community services to maintain the continuity of care. The behavior dimension was measured through the physical health QOL indicator, which covered elements such as pain, fatigue, sleep, activities of daily living, and substance dependence. Therefore, psychological/physical health was not a direct outcome of the case management. With regard to the social dimension, the social relationship’s QOL covers the personal relationships. No intervention was made on this dimension by the case management team. The second reason for the absence of significant impact on those dimensions can be explained by the study’s length. Twelve months can be enough for a brief mental health intervention to impact patents’ QOL. However, the case management intervention took place during the first 5 months. And establishing a network between patients and health providers to give the more suitable psychological help to the patient can take some time before accessing to a mental health intervention. In the remaining months of the study, the impact of the intervention on some psychological elements may have been too short to be effectively caught by quantitative measures as the WHOQOL. Therefore, studies with a longer follow-up are needed to investigate whether the case management affected these three dimensions of QOL. Additionally, case management focusing directly on these dimensions should be developed. The purpose of the case management was to improve frequent users’ health and QOL [9, 22] by providing more adequate comprehensive care measures. However, the effectiveness of the case management focused on reducing the number of visits or of number of hospitalization. Research has shown that ED use and its associated costs were significantly [22, 25, 27, 48] or marginally [35, 49] reduced. However, two systematic reviews [50, 51] pointed out that future ED visit reduction interventions needed rigorous evaluation. This last point was to be reinforced by considering the QOL level of this vulnerable population to determine the most global, long-term, and appropriate programs.

Our study had some limitations. First, the number of dropouts was significant as one-fifth of the participants included at baseline died or did not participate in the whole study. However, this was balanced between the control and intervention groups. Participants who died during the study had the same level of QOL as patients who completed the whole study. Also, dropout patients (those who left the study) had the same level of QOL at baseline except for the environment QOL dimension. As the intervention had a significant impact on the environment QOL dimension and as this group was significantly more vulnerable in this dimension, it seems particularly important to identify those patients to enable them to benefit from interventions. The second limitation concerns the representativeness of the frequent ED users. Our sample is constituted of the “less vulnerable” patients, as 75% of the sample at baseline had between five and six ED visits. This study should be replicated in larger samples and with a larger number of ED with potentially more vulnerable frequent users. Finally, to better understand the evolution of the self-reported measures of QOL, an additional qualitative approach through interview would have provided better insights into the study’s results.

## Conclusions

This is the first study to assess the level of QOL of frequent ED visitors using a RCT to measure the impact of a case management intervention. Health care practitioners should be aware of the possible positive impact of case management intervention on patients’ environment QOL in short-term interventions. QOL is an important indicator when analyzing a vulnerable population, such as frequent ED users. However, further researches are needed to confirm our findings, and to develop and evaluate case management interventions for frequent ED users’ QOL as it is important to consider the social and medical complexity and the vulnerability of this population in a long-term perspective. Therefore, from a research perspective, case managements for frequent ED users should be tested in other settings and be part of multicenter studies and QOL should be assessed through mixed methods. This would add robust evidence of the potential benefits of such interventions. From a clinical perspective, case management with a focus on QOL should also benefit other groups of vulnerable patients, such as psychiatric patients or prisoners on release. For the last group, case management would help rehabilitation and a potential crucial outcome would be recidivism. We encourage future research on this specific question.

## Abbreviations

ED: Emergency Department
QOL: Quality of life
RCT: Randomized controlled trial
SD: Standard deviation
